# Supplementary material for: The Development of a 10-Item Ventilator-Associated Pneumonia Care Bundle in the General Intensive Care Unit of a Tertiary Hospital in Vietnam: Lessons Learned
Source: Healthcare (Basel). 2025 Feb 20;13(5):443. doi: 10.3390/healthcare13050443 (PMC11899602; doi:10.3390/healthcare13050443)
Supplement: Supplementary file 1 [file healthcare-13-00443-s001.zip › healthcare-3430882-supplementary.pptx]

## Slide 1
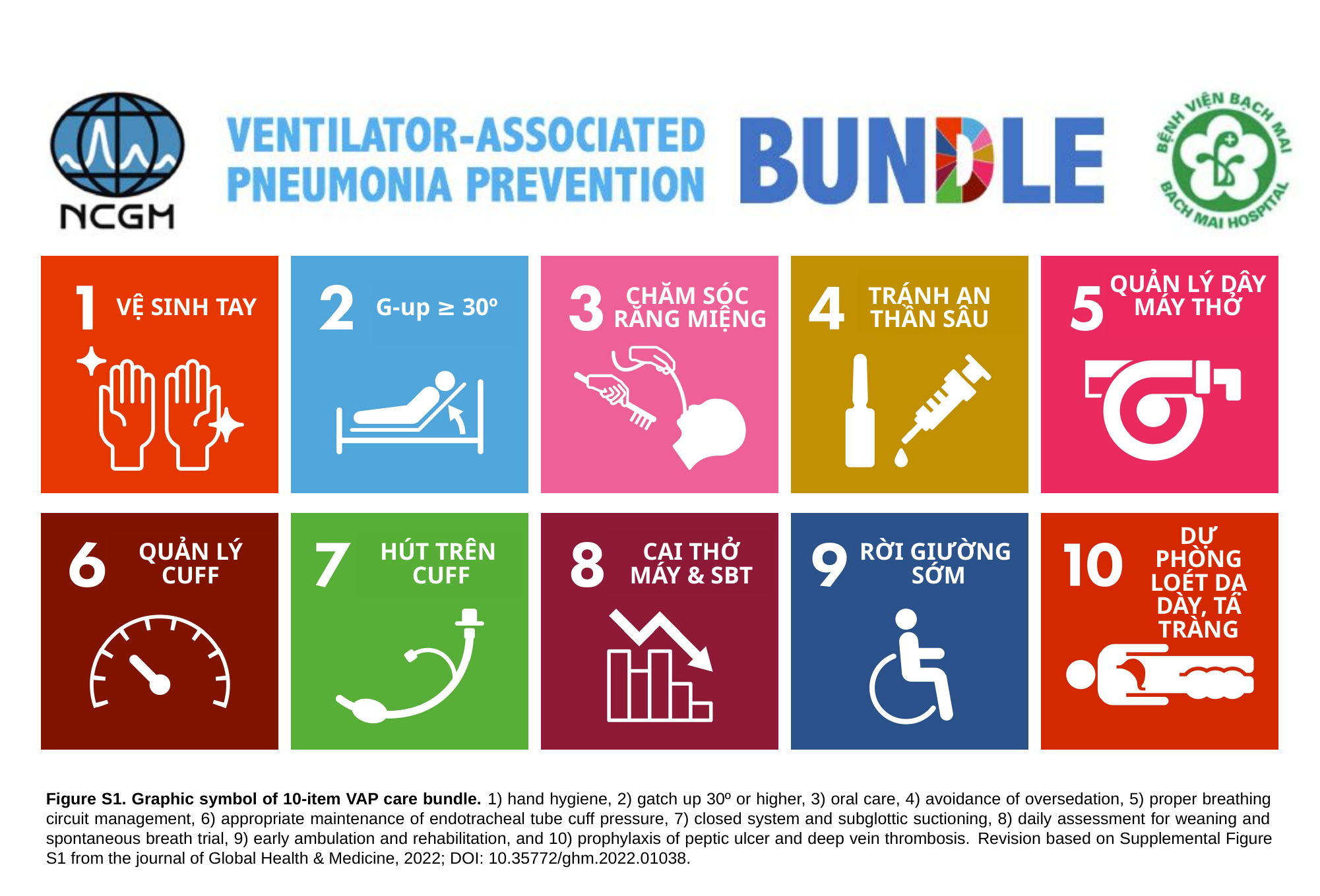

1
2
3
4
5
QUẢN LÝ DÂY MÁY THỞ
CHĂM SÓC
RĂNG MIỆNG
TRÁNH AN
THẦN SÂU
VỆ SINH TAY
G-up ≥ 30º
DỰ PHÒNG LOÉT DẠ DÀY, TÁ TRÀNG
QUẢN LÝ
CUFF
HÚT TRÊN
CUFF
CAI THỞ
MÁY & SBT
RỜI GIƯỜNG
SỚM
Figure S1. Graphic symbol of 10-item VAP care bundle. 1) hand hygiene, 2) gatch up 30º or higher, 3) oral care, 4) avoidance of oversedation, 5) proper breathing circuit management, 6) appropriate maintenance of endotracheal tube cuff pressure, 7) closed system and subglottic suctioning, 8) daily assessment for weaning and spontaneous breath trial, 9) early ambulation and rehabilitation, and 10) prophylaxis of peptic ulcer and deep vein thrombosis. Revision based on Supplemental Figure S1 from the journal of Global Health & Medicine, 2022; DOI: 10.35772/ghm.2022.01038.
